# Long non-coding RNA-NONMMMUT004552.2 regulates the unloading-induced bone loss through the miRNA-15b-5p/Syne1 in mice

**DOI:** 10.1038/s41526-024-00382-8

**Published:** 2024-03-23

**Authors:** Zheng Zhang, Yu Jing, Ang Zhang, JiShan Liu, Heming Yang, Xiaotong Lou, Liyan Xu, Min Liu, Yikun Zhang, Jianwen Gu

**Affiliations:** 1 Department of Medical Engineering, PLA Strategic Support Force Characteristic Medical Center, Beijing, 100101 China; 2 Department of Haematology, The Fifth Medical Centre of Chinese PLA General Hospital, Beijing, 100071 China; 3 Department of Hematology, PLA Strategic Support Force Characteristic Medical Center, Beijing, 100101 China; 4 School of Biological Science and Medical Engineering, Beihang University, Beijing, 100191 China; 5 Department of General Surgery, PLA Strategic Support Force Characteristic Medical Center, Beijing, 100101 China; 6 Department of Research, PLA Strategic Support Force Characteristic Medical Center, Beijing, 100101 China; 7 Department of Blood Transfusion, PLA Strategic Support Force Characteristic Medical Center, Beijing, 100101 China; 8 Department of Neurosurgery, PLA Strategic Support Force Characteristic Medical Center, Beijing, 100101 China

**Keywords:** Molecular biology, Cell biology

## Abstract

Exercise-induced mechanical loading can increase bone strength whilst mechanical unloading enhances bone-loss. Here, we investigated the role of lncRNA NONMMUT004552.2 in unloading-induced bone-loss. Knockout of lncRNA NONMMUT004552.2 in hindlimb-unloaded mice caused an increase in the bone formation and osteoblast activity. The silencing of lncRNA NONMMUT004552.2 also decreased the osteoblast apoptosis and expression of Bax and cleaved caspase-3, increased Bcl-2 protein expression in MC3T3-E1 cells. Mechanistic investigations demonstrated that NONMMUT004552.2 functions as a competing endogenous RNA (ceRNA) to facilitate the protein expression of spectrin repeat containing, nuclear envelope 1 (Syne1) by competitively binding miR-15b-5p and subsequently inhibits the osteoblast differentiation and bone formation in the microgravity unloading environment. These data highlight the importance of the lncRNA NONMMUT004552.2/miR-15b-5p/Syne1 axis for the treatment of osteoporosis.

## Introduction

Osteoporosis is defined as a skeletal disorder characterized by compromised bone strength predisposing a person to an increased risk of fracture^1^. Osteoporosis induced fractures represent a major burden to healthcare systems and are a major cause of mortality^2^. Involutional or senile osteoporosis causes loss of both cortical and trabecular bone, whereas post-menopausal and steroid-induced osteoporosis have the greatest impact on trabecular bone^3^. Calcium plus vitamin D, estrogen replacement therapy, calcitonin, and etidronate are agents currently available for treatment of osteoporosis^4^. Current anti-osteoporosis treatments decrease the occurrence of fractures, but bone necrosis, hypercalcemia and thromboembolic disease represent major side-effects^2^. HU (Hindlimb-unloaded) animal models have been used to study unloading-related bone loss^5,6^.

Mechanical loads influence the development of the musculoskeletal system. During extended bed-rest and long-duration space flights, prolonged mechanical unloading by microgravity (MG) promotes osteoporosis^7–9^. Bone can be reabsorbed and rebuilt by osteoblasts^10,11^ in a manner regulated by hormones, cytokines and mechanical stimuli^5^. Loss of the capacity of osteoblasts to transfer mechanical loading to biochemical signals contributes to disease pathogenesis^12–15^. LncRNAs as known regulators of gene expression have been extensively studied in the context of osteogenesis^16–18^ and unloading-induced bone loss^2,9,19,20^. For example, lncRNA NONMMUT002009 promoted interacted with miR-139-3p and was able to regulate the osteoblast differentiation and apoptosis under simulated microgravity through its target gene ELK1^9,14^. LncRNA Neat1 promotes osteoclastogenesis through sponging miR-7^21^. Neat1 is crucial for the activity of osteoblasts in the presence of mechanical stimulation and prevents bone-loss and osteoporosis^22^. Through the previous analysis of sequencing from our research, we found that in HU mice under simulated microgravity unloading, there was a highly expressed lncRNA (lncRNA NONMMUT004552.2) related to bone metabolism, and at the same time, we also found a highly expressed gene (Syne1). However, whether NONMMUT004552.2 and Syne1 in the osteogenic region can regulate bone loss after mechanical unloading, and how they play their roles still need to be further explored.

In this study, we investigated the function of lncRNA NONMMUT004552.2 (Table S1) in HU mice, and confirmed that lncRNA NONMMUT004552.2 decreased the bone formation and osteoblast activity in HU mice. lncRNA NONMMUT004552.2 could promote the osteoblast apoptosis and inhibit osteoblast mineralization in vitro. In addition, The lncRNA NONMMUT004552.2 could regulate Syne1 expression by interacting with miR-15b-5p. The lncRNA NONMMUT004552.2 partly promoted apoptosis and reduced differentiation in MC3T3-E1 cells in a manner partially dependent on miR-15b-5p and Syne1 in a microgravity unloading environment. These findings highlight the lncRNA NONMMUT004552.2 as a therapeutic target for the treatment of osteoporosis and pathological osteopenia.

## Results

### Knockout of lncRNA NONMMUT004552.2 alleviates the loss of osteoblast activity and bone formation in hindlimb-unloaded (HU) mice

**Fig. 1:**
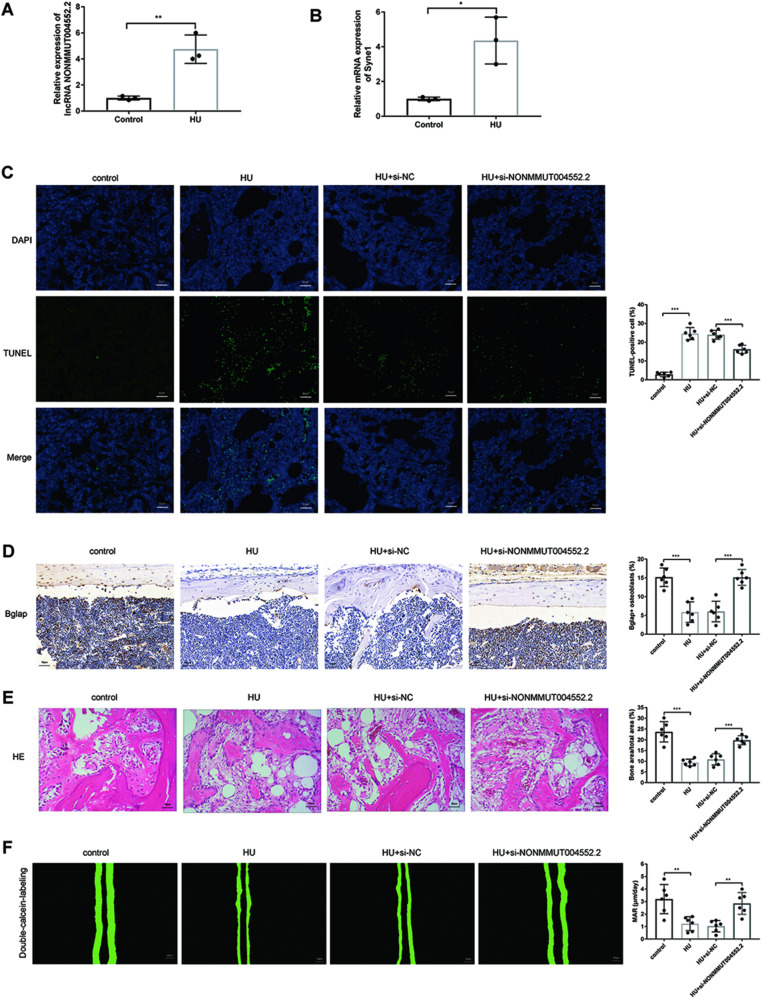
Knockout of lncRNA NONMMUT004552.2 counteract the loss of bone formation in hindlimb-unloaded (HU) mice. **A** qRT-PCR analysis of LncRNA NONMMUT004552.2 level in HU mice. **B** qRT-PCR analysis of Syne1 level in HU mice. **C** Representative TUNEL images of the distal femurs in the indicated groups. TUNEL staining of apoptotic cells is green, whereas DAPI staining is blue. Scale bar = 50 µm; *N* = 6. **D** Representative images of Bglap staining of the distal femurs of mice. Scale bar = 50 µm; *N* = 6. **E** Representative H&E staining of the distal femurs of mice in the indicated groups. Statistical analysis of the histological parameters of bone area per total area (B.Ar per T.Ar) in the proximal region via H&E staining (Scale bar = 50 µm; *N* = 6). **F** Representative images of new bone formation assessed by double calcein labeling. Scale bar = 50 µm.

To identify the mechanosensitive lncRNAs in osteoblasts, we performed RNA sequencing of the primary osteoblasts from mice exposed to simulated microgravity (MG) unloading conditions. We found that lncRNA NONMMUT004552.2 (*P* < 0.01) and Syne1 (*P* < 0.05) were highly expressed by qRT-PCR in HU mice compared with control group (Figs. 1A and 1B). To further investigate how they interact in bone metabolism, hindlimb-unloaded (HU) mouse models are the most commonly used in vivo models for inducing bone loss due to unloading. After 21 days of hindlimb unloading, a marked increase in the number of TUNEL+ cells were observed in the mice distal femurs of the HU group compared to the control group. Fewer apoptotic cells were observed in the HU+si-NONMMUT004552.2 group compared to HU + si-NC mice (*P* < 0.001, Fig. 1C). The number of Bglap+ osteoblasts were significantly lower in mouse femurs of the HU group compared to the control group. Bglap+osteoblasts in the HU+si-NONMMUT004552.2 group were higher than HU+si-NC mice (*P* < 0.001, Fig. 1D). Compared to the control group, HU mice showed a reduced total bone area following H&E analysis. The administration of si-NONMMUT004552.2 significantly restored the bone area per total area (*P* < 0.001, Fig. 1E). Bone formation (the mineral apposition rate (MAR)) was markedly lower in the HU group compared to control mice. Bone formation was higher in the si-NONMMUT004552.2-treated HU groups compared to HU + si-NC mice (*P* < 0.001, Fig. 1F). Collectively, these data show that lncRNA NONMMUT004552.2 facilitates the loss of bone formation and osteoblast activity in HU mice.

### LncRNA NONMMUT004552.2 promotes osteoblasts apoptosis and suppresses mineralization in osteoblasts

**Fig. 2:**
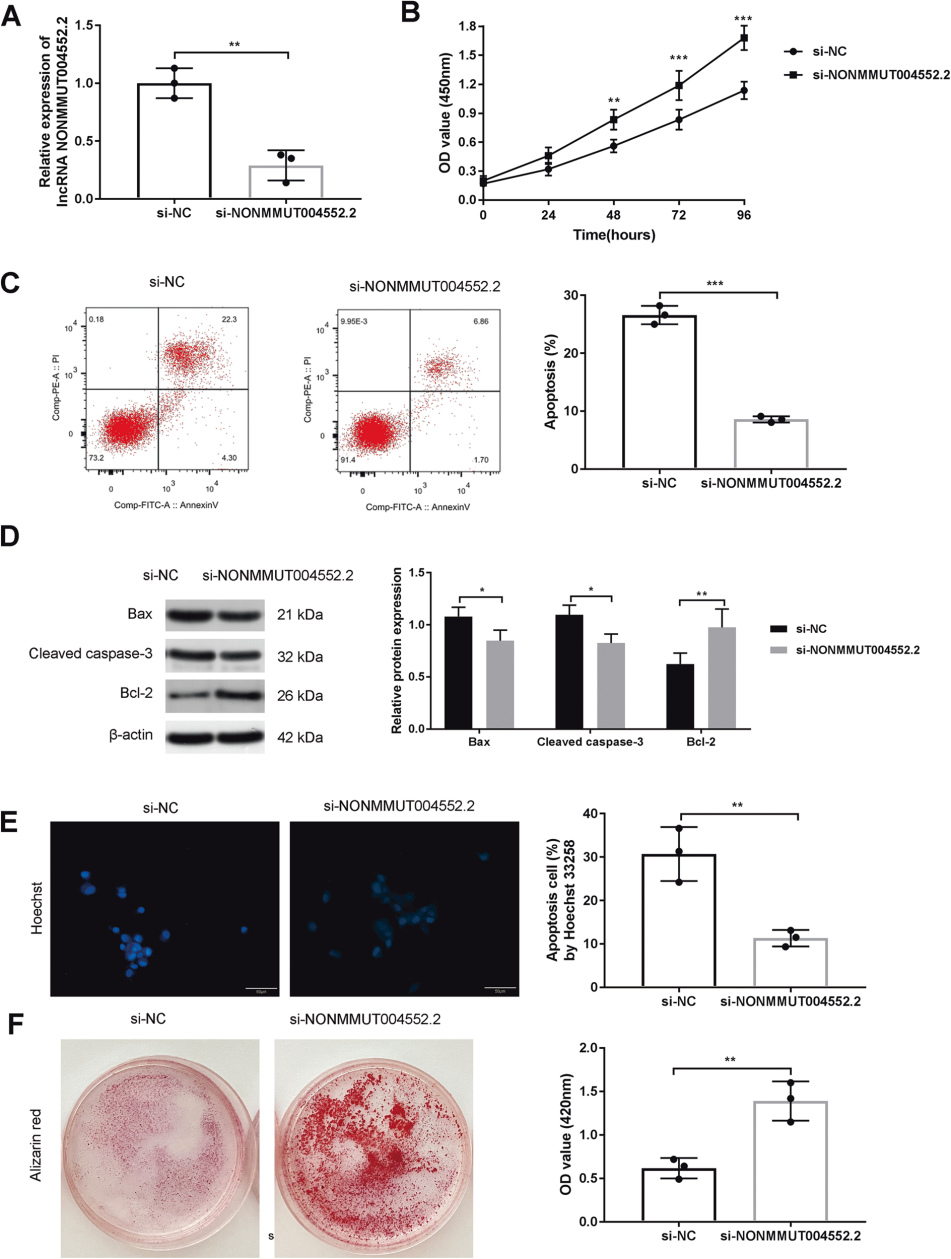
LncRNA NONMMUT004552.2 promote osteoblast apoptosis and inhibits osteoblast mineralization. **A** The expression of LncRNA NONMMUT004552.2 were measured by qRT-PCR in MC3TC-E1 cells. **B** Cell proliferation of MC3TC-E1 was examined by CCK8 assay. **C** Flow cytometry of osteoblasts stained with Annexin V-FITC and PI. **D** Bax, Bcl-2, and cleaved caspase-3 expression in osteoblasts. **E** Representative Hoechst 33258 staining in osteoblasts (Scale bar = 50 µm) and quantification of apoptosis cells. **F** Representative Alizarin red staining in osteoblasts and quantification of matrix mineralization. *P < 0.05, **P < 0.01 and ***P < 0.001.

We further explored the effects of lncRNA NONMMUT004552.2’s on in vitro osteoblast function. MC3T3-E1 cells were transfected with si-NONMMUT004552.2, or si-NC, and were cultured in the osteogenic medium. The result of Qrt-PCR showed that the expression of LncRNA NONMMUT004552.2 was decreased in si-NONMMUT004552.2 group, when compared to the si-NC group (*P* < 0.01, Fig. 2A). CCK-8 assay showed us that si-NONMMUT004552.2 markedly increased the proliferation of MC3T3-E1 compared to si-NC group (P < 0.001, Fig. 2B). In addition, NONMMUT004552.2 silencing markedly decreased the number of apoptotic osteoblasts (*P* < 0.001, Fig. 2C) and increased the expression of Bcl-2. The expression of cleaved caspase-3 and Bax was also lower in silenced cells (*P* < 0.05, *P* < 0.01, Fig. 2D). Hoechst 33258 staining confirmed the presence of apoptotic nuclei was higher in si-NONMMUT004552.2 transfected cells (Fig. 2E, *P* < 0.01). Alizarin red staining revealed higher levels of matrix mineralization in si-NONMMUT004552.2 group compared to si-NC group (Fig. 2F, *P* < 0.01). These data suggested that lncRNA NONMMUT004552.2 promotes osteoblast apoptosis and inhibits osteoblast mineralization.

### LncRNA NONMMUT004552.2 regulates Syne1 expression depends on miR-15b-5p

**Fig. 3:**
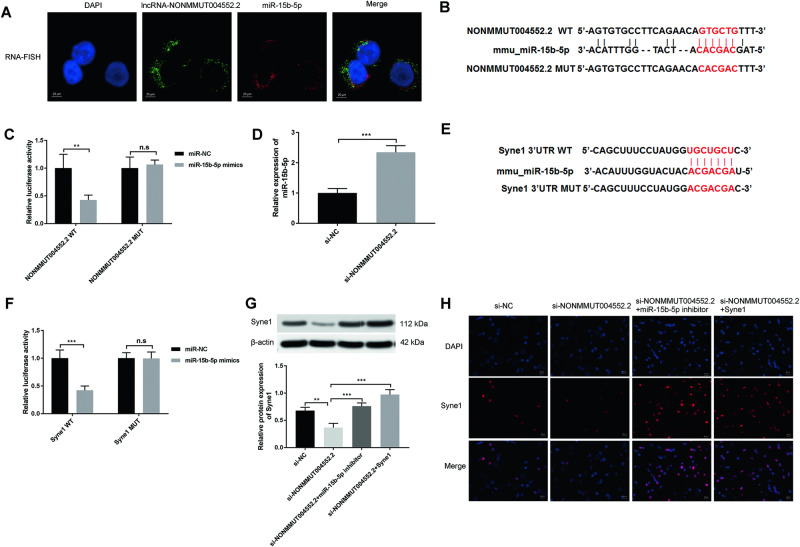
LncRNA NONMMUT004552.2 interacts with miR-15b-5p to regulate Syne1 expression. **A** Co-localization of lncRNA NONMMUT004552.2 and miR-15b-5p in MC3T3-E1 determined by RNA fluorescence in situ hybridization (Scale bar = 25 µm). **B** The putative sequences of miR-15b-5p and lncRNA NONMMUT004552.2 with binding sites. **C** miR-15b-5p significantly inhibited luciferase activity of wild type reporter for lncRNA NONMMUT004552.2. **D** qRT-PCR analysis of miR-15b-5p level in si-NONMMUT004552.2 transfected-MC3T3-E1 cells. **E** The putative target region analysis was performed for Syne1 3’ UTR by miR-15b-5p seed. **F** The relative luciferase activity of luciferase reporters showed that miR-15b-5p significantly inhibited luciferase activity of wild type reporter for Syne1 in MC3TC-E1 cells. **G** Western blot was conducted to measure the protein level of Syne1 expression in osteoblasts (MC3T3-E1) co-transfected with si-NONMMUT004552.2; si-NONMMUT004552.2+miR-15b-5p inhibitor; or si-NONMMUT004552.2+Syne1. **H** Immunostaining of Syne1 in transfected osteoblasts (MC3T3-E1). (Scale bar = 20 µm). *P < 0.05, **P < 0.01.

As the function of lncRNA NONMMUT004552.2 on osteogenesis during unloading was confirmed both in vitro and in vivo, we further explored the mechanisms involved in this process. RNA fluorescence in situ hybridization (RNA-FISH) of lncRNA NONMMUT004552.2 and miR-15b-5p showed high levels of colocalization in the cytoplasm of MC3T3-E1 osteoblasts (Fig. 3A). Thus, we speculated that NONMMUT004552.2 may act as a ceRNA that binds to microRNAs. Bioinformatics analysis (https://cm.jefferson.edu/rna22/Interactive/) revealed that the lncRNA NONMMUT004552.2 sequence contains several putative binding sites for microRNAs, and miR-15b-5p has been found to be a positive regulator for osteoblast differentiation^23^. So we selected miR-15b-5p involved in osteoblast differentiation for further study (Fig. 3B). Next, the binding between miR-15b-5p and lncRNA NONMMUT004552.2 was further validated by dualluciferase reporter assays. We found that miR-15b-5p mimic significantly reduced the luciferase activity of the reporter (*P* < 0.01, Fig. 3C). The result of Qrt-PCR demonstrated that the level of miR-15b-5p was markedly increased in MC3T3-E1 cells after transfected with si-NONMMUT004552.2 (*P* < 0.001, Fig. 3D). Considering that mmu-miR-15b-5p was predicted to target Syne1 (Fig. 3E) by TargetScan (https://www.targetscan.org/cgi-bin/targetscan/mmu_72/view_gene.cgi?rs=ENSMUST00000095899.3&taxid=10090&members=&showcnc=0&shownc=0&showncf1=&showncf2=&subset=1), we constructed luciferase reporters containing either the WT Syne1 3’-UTR or the Syne1 3’UTR with mutated (MUT) miR-15b-5p-binding sites in MC3T3-E1 cells, and found that miR-15b-5p substantially inhibited the luciferase reporter activity of the Syne1 3’UTR, but not the Syne1 3’UTR MUT (*P* < 0.001, Fig. 3F). Our results confirmed that lncRNA NONMMUT004552.2 can act as a ceRNA for binding to miR-15b-5p, and Syne1 is the target of miR-15b-5p. The further study was conducted to explore whether the lncRNA NONMMUT004552.2 can regulate the expression of the miR-15b-3p-targeted gene Syne1 in MC3T3-E1 cells. as indicated by western blot and indirect immunofluorescence assays (Fig. 3G, H), co-transfection of miR-15b-5p inhibitor (or Syne1 experssion) with si-NONMMUT004552.2 partially alleviated the si-NONMMUT004552.2-mediated decrease in Syne1 expression (*P* < 0.01; *P* < 0.001). Collectively, these data suggest that lncRNA NONMMUT004552.2 interacts with miR-15b-5p to regulate Syne1 expression in MC3T3-E1 cell.

### lncRNA NONMMUT004552.2 promotes the apoptosis in MC3T3-E1 cells under unloading conditions depends on miR-15b-5p/Syne1

**Fig. 4:**
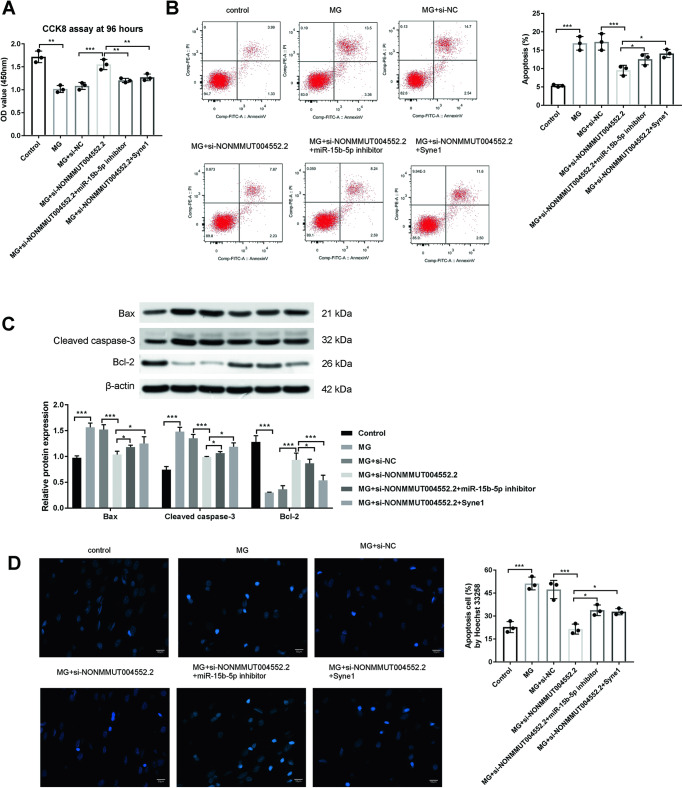
LncRNA NONMMUT004552.2 promotes apoptosis in MC3T3-E1 cells depends on miR-15b-5p/Syne1 under MG unloading conditions. **A** CCK-8 assay showed the proliferation of different transfected-MC3T3-E1 cells at 96 h. **B** Flow cytometry in osteoblasts stained with Annexin V-FITC and PI. **C** Bax, Bcl-2, and cleaved caspase-3 expression in osteoblasts. **D** Hoechst 33258 staining (Scale bar = 50 µm). *P < 0.05, **P < 0.01, and ***P < 0.001.

Since lncRNA NONMMUT004552.2 interacted with miR-15b-5p, and Syne1 was the target of miR-15b-5p, we examined whether miR-15b-5p and Syne1 could participate in the role of lncRNA NONMMUT004552.2 on osteoblasts under MG. We transfected MC3T3-E1 cells with si-NONMMUT004552.2 si-NONMMUT004552.2+miR-15b-5p inhibitor or si-NONMMUT004552.2+Syne1 for 12 h and then cultured in MG unloading environment for 2 d. CCK-8 assay showed that the cell proliferation was decreased in the MG group compared to control cells (*P* < 0.001). In the MG + si-NONMMUT004552.2 group, the cell proliferation of MC3T3-E1 cells was significantly increased in response to MG+si-NC (*P* < 0.001), which was restored following the transfection of miR-15b-5p inhibitor or Syne1 (*P* < 0.05; *P* < 0.01) **(**Fig. 4A). Moreover, MC3T3-E1 cell apoptosis increased in the MG group compared to control cells. In the MG+si-NONMMUT004552.2 group (*P* < 0.001), the number of apoptotic MC3T3-E1 cells significantly decreased in response to MG, which was restored following the transfection of miR-15b-5p inhibitor or Syne1 (*P* < 0.05) **(**Fig. 4B). Compared to cells in the MG + si-NC group, the MG+si-NONMMUT004552.2 group showed the decreased levels of apoptosis-related protein expression (Bax and cleaved caspase-3) and increased Bcl-2 expression (*P* < 0.001), While, miR-15b-5p inhibitor+si-NONMMUT004552.2 or Syne1+si-NONMMUT004552.2 transfection could increase levels of Bax and cleaved caspase-3 and decreased Bcl-2 expression when compared to the MG+si-NONMMUT004552.2 group (*P* < 0.05; *P* < 0.001 Fig. 4C). Hoechst 33258 staining showed a significant reduction in MG+si-NONMMUT004552.2 apoptotic nuclei that increased following the transfection of si-NONMMUT004552.2+miR-15b-5p inhibitor or si-NONMMUT004552.2+Syne1 (*P* < 0.05; *P* < 0.001; Fig. 4D). These results confirmed that lncRNA NONMMUT004552.2 increased the osteoblast apoptosis by interacting with miR-15b-5p/Syne1.

### LncRNA NONMMUT004552.2 reduces differentiation in MC3T3-E1 cells partially depends on miR-15b-5p/Syne1 under unloading condition

**Fig. 5:**
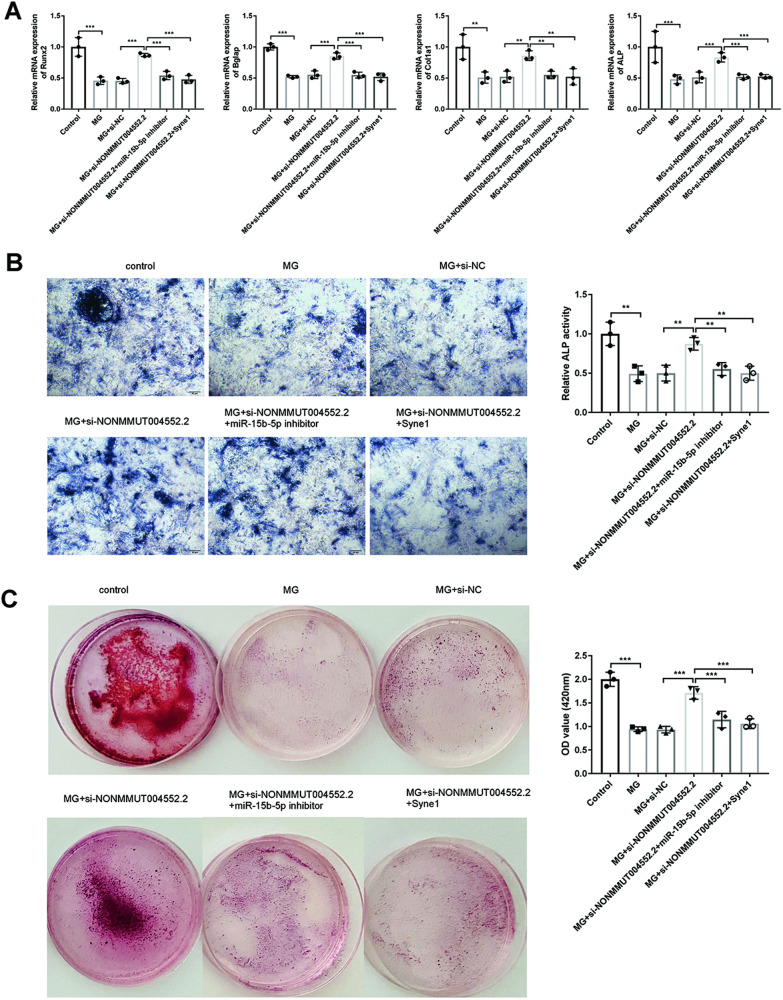
LncRNA NONMMUT004552.2 suppresses osteoblast differentiation in MC3T3-E1 cells depends on miR-15b-5p/Syne1 under MG unloading conditions. **A** mRNA expression of osteoblast marker genes (Runx2, Bglap, Col1a1, and ALP; normalized to GAPDH). **B** Representative images of ALP staining in MC3T3-E1 cells and ALP activity determination. **C** Representative images of alizarin red staining in MC3T3-E1 cells. Relative mineralization was quantified using Image J. *P < 0.05, **P < 0.01, and ***P < 0.001.

Qrt-PCR assay investigated that the mRNA levels of osteogenic genes, such as Runx2, Bglap, Col1a1, and ALP were decreased in the unloading condition. si-NONMMUT004552.2 transfection also markedly upregulated the expression of Runx2, Col1a1, Bglap, and ALP, whereas miR-15b-5p inhibitor or Syne1 overexpression reduced the upregulation induced by the si-NONMMUT004552.2 in the unloading environment (Fig. 5A). si-NONMMUT004552.2 transfection also markedly upregulated the expression of Runx2, Col1a1, Bglap, and ALP at the mRNA level, which were inhibited by the co-transfection of Syne1 (*P* < 0.05; *P* < 0.01; *P* < 0.001) (Fig. 5A). osteoblastic differentiation, as assessed by the alkaline phosphatase (ALP) activity, ALP staining. ALP activity and ALP staining showed the similar trend (*P* < 0.01, Fig. 5B). Matrix mineralization significantly increased in response to si-NONMMUT004552.2 under unloading condition, and decreased following the co-transfection of miR-15b-5p inhibitor or Syne1 (*P* < 0.001, Fig. 5C). These results confirmed that NONMMUT004552.2 suppressed the osteogenic differentiation by interacting with miR-15b-5p and targeting Syne1.

## Discussion

Bone loss is regulated by various cytokines, hormones and miRNAs^2,24^. However, the role of lncRNAs in unloading-induced bone loss is less well understood. In this study, we reveal a mechanism by which the lncRNA NONMMUT004552.2 inhibits the osteoblast differentiation and promotes the bone loss following mechanical unloading in mice. The silencing of lncRNA NONMMUT004552.2 was found to inhibit apoptosis, promote osteoblast mineralization in MC3T3-E1 osteoblast. we further confirmed that the lncRNA NONMMUT004552.2 can increase the apoptosis and decrease the mineralization of MC3T3-E1 cells in the MG unloading environment, and that it can regulate Syne1 expression in a manner partially dependent on miR-15b-5p. This highlights the lncRNA NONMMUT004552.2/miR-15b-5p/Syne1 axis as a therapeutic target during osteoporosis.

Osteoblasts are key mediators of bone formation, responsible for the synthesis, secretion and mineralization of the bone matrix, allowing bone to grow and rebuild^25^. Increased osteoblast differentiation and the prevention of osteoblast apoptosis are important mechanisms in the pathogenesis of osteoporosis^26,27^. Multiple lncRNAs regulate bone formation and osteoblast differentiation^28^. LncRNA MEG3 activates the transcription of BMP4 through the disassociation of SOX2, which promotes osteoblast differentiation in MSC^29^. LncRNA Bmncr alleviates fat accumulation in bone marrow cells and prevents bone loss by facilitating the assembly of the RUNX2/PPARG and the TAZ transcriptional complex during aging^30^. LncRNA PGC1β-OT1 stimulates progenitor cell osteoblast differentiation, whilst LncRNA OGRU promotes bone formation^2^. To date, lncRNAs and circular RNAs, have been thoroughly characterized as ceRNAs for microRNA binding^31^. Here, we show that lncRNA NONMMUT004552.2 localizes to the cytoplasm of osteoblasts and promotes unloading-associated bone loss through its interaction with miR-15b-5p.

Previously, it has been reported that miR-15b-5p is a a positive regulator for osteoblast differentiation, In human BMSCs, miR-15b promotes the osteoblasts differentiation and shows a high amount of ALP and type I collagen. This process targets Smurf1 which protects Runx2 from degradation^23^. In addition, The expression level of miR-15b is also regarded as one of the criteria for the osteogenic evaluation^32^. In this study, the experiments strongly suggested that miR-15b-5p is unloading-sensitive, and that its levels can be decreased by lncRNA NONMMUT004552.2 during unloading. Furthermore, we demonstrated that Syne1 is the target of miR-15b-5p.

Syne1, located on chromosome 6, encodes a spectrin repeat containing protein expressed in skeletal and smooth muscle, and peripheral blood lymphocytes, that localizes to the nuclear membrane^33,34^. It plays a role in cardiomyocyte and skeletal muscle development, particularly in DNA damage response pathways^35^. The SYNE1 expression also impacted stem cell pluripotency and differentiation capacity^36^. Most importantly, inhibiting the expression of Syne1 in rat mesenchymal stem cells decreases cell proliferation and increases apoptosis^37^. However, in the present study, we found that Syne1 had highly expressed in the HU mice. Interestingly, lncRNA NONMMUT004552.2 and Syne1 show the same expression patterns. Additionally, TargetScan and luciferase activity assays confirmed the binding between Syne1 and miR-15b-5p. Moreover, Syne1 expression was down-regulated by miR-15b-5p. miR-15b-5p inhibitor or Syne1 overexpression reduced the upregulation of osteoblast differentiation induced by the si-NONMMUT004552.2 in unloading condition. Thus, our results revealed that lncRNA NONMMUT004552.2 promotes bone loss by sponging miR-15b-5p, which subsequently regulates Syne1 expression.

In summary, our findings show that lncRNA NONMMUT004552.2 increases the apoptosis and inhibits osteoblasts differentiation during mechanical unloading-induced bone loss. lncRNA NONMMUT004552.2, which is a critical regulator of osteoblast function, and promotes the bone loss by functioning as a miR-15b-5p sponge to stimulate Syne1 expression. represents the important therapeutic target for osteoporosis in response to mechanical loading.

## Methods

### HU mouse models

C57BL/6 J mice (6-month-old; Male) were purchased from the Beijing Vital River Laboratory (Beijing, China) and housed in a 21 °C incubator with a 12 h light/dark cycle and free access to food and water. Mice were were randomly divided into the following groups (*n* = 6 per-group): [1] Control; [2] HU; [3] HU+si-NONMMUT004552.2; [4] HU+si-NC. The hindlimb unloading (HU) model is one of the models of bone loss caused by mechanical unloading as previously reported^14,38^. Briefly, The mouse’s tail was attached to a piece of surgical tape that formed a loop close to the end of the tail. Three strips of elastic adhesive bandage were used to secure the surgical tape that was placed on the tail.The mouse’s forelimbs had full access to the entire cage. HU mice were hung from the top of the cage by the tail at a 30°angle with only the forelimbs touching the floor for 4 weeks, which allowed them to move and access food and water freely. The control group was mice without suspended tails in cage. Before HU, we encapsulated an osteogenic siRNA that target lncRNA NONMMUT004552.2 (si-NONMMUT004552.2) to the bone formation zone, mice in the experimental group were injected with si-NC, and si-NONMMUT004552.2 plasmids via the caudal vein with 2 mg kg^−1^ plasmids every day for 3 consecutive days using the (AspSerSer) 6-liposome delivery system^9,19^. After 3-weeks of tail suspension, mice were euthanized and the bilateral femurs and tibiae harvested. No mice died during the procedure. All protocols were approved by the Animal Ethics Committee guidelines of Beijing Viewsolid Biotechnology Co. Ltd. (VS2602A12318).

### TUNEL assays

Tibias from HU mice were fixed in 4% paraformaldehyde, decalcified, paraffin embedded and stained using the DeadEnd™ Fluorometric TUNEL System. Then, slides were stained with the DeadEnd™ Fluorometric TUNEL System (Promega, USA), and imaged using a fluorescent microscope (Evos FL Cell Imaging System; Life Technologies). DNase treatments were used as positive controls (green) for TUNEL staining.

### Immuno- and histological analysis

Femurs from HU mice were fixed in 4% paraformaldehyde, decalcified using 10% ethylenediaminetetraacetic acid (EDTA, Beyotime Biotechnology, Shanghai, China) and embedded in paraffin. For immunohistochemistry, sections were dewaxed, blocked in 5% goat serum and stained for 24 h at 4 °C with primary antibody against the following specific protein Bglap (1:50; ab93876, Abcam). Subsequently, diaminobenzidine and hematoxylin were used to detect immunoreactivity. For histological analysis, Sections were stained with H & E and detected using diaminobenzidineused. Slides were imaged on a FV1000 confocal microscope (Olympus, Japan).

### Double calcein labeling assay

To evaluate the dynamic indexes of bone formation, mice were intraperitoneally injected with 8 mg/kg calcein (body weight, Sigma, St. Louis, MO, USA) 10 days and 3 days before sacrifice. Fibias were harvested, fixed in 4% paraformaldehyde for 2 days and embedded in polymethylacrylate. Samples were cut into 3 sections (thickness ~50 μm) using a hard tissue slicing machine (SP1600, Leica, Germany) in the dark. Slides were imaged following double-calcein labeling by confocal microscopy (LSM800, ZEISS, Germany). The distance between two fluorescence-labeled lines as measured three times with Image J software was used to evaluate the mineral apposition rate (MAR) of bone.

### Cell culture

Mouse MC3T3-E1 osteoblasts were purchased from the Chinese Academy of Sciences (China). Cells were cultured at 5% CO_2_, 95% humidity at 37 °C in α-MEM supplemented with 10% FBS and 1% penicillin or streptomycin. Cells at passage 8–12 were induced with osteogenic media containing 10 mM β-glycerophosphate, 100 nM dexamethasone and 50 μM ascorbic acid. Assayed were performed on a minimum of 3 occasions.

### Cell culture under microgravity (MG) unloading condition

A two-dimensional (2D) clinostat was used to simulate the effects of MG for cells cultured. Briefly, MC3T3-E1 cells (1 × 10^5^ cells per well) were plated on the cover glasses in a 6-well plate. After ~8 h, cells adhered to the walls, and the cover glasses were inserted into a chamber filled with culture medium. The distance between the cover glasses and the rotating axis of the chamber was 12.5 mm. Then, the caps of the chamber were tightened after all bubbles were gently removed. Finally, the chambers were placed into a clinostat and rotated around a horizontal axis at 24 rpm. The vertical rotation group served as the control group. The whole process of cell culture in the MG unloading environment was carried out at 37 °C. Experiments were carried out according to the previous research^9^.

### Cell transfection

LncRNA NONMMUT004552.2 siRNA (si-NONMMUT004552.2) and siRNA negative control (si-NC), were designed and synthesized by GenePharma (Shanghai, China). Full length Syne1 genomic DNAs were inserted into pcDNA3.1 vectors to establish Syne1 overexpression vector (Syne1). MC3T3-E1 cells were transfected with si-NONMMUT004552.2, lncRNA NONMMUT004552.2 wild-type reporters (NONMMUT004552.2 WT), lncRNA NONMMUT004552.2 mutant-type reporters (NONMMUT004552.2 MUT), miR-15b-5p mimic and miR-15b-5p inhibitor or their corresponding negative controls, Syne1 and Syne1 wild-type reporters (Syne1 WT), Syne1 mutant-type reporters (Syne1 MUT), purchased from GenePharma (Shanghai, China) using Lipofectamine 3000 as per the manufacturer’s recommendations (Thermo Fisher Scientific, Waltham, MA, USA).

### Cell counting kit-8 (CCK8) assay

CCK-8 assay was performed to evaluate the viability of osteoblasts. MC3T3-E1 cells were plated in 96-well plates at a density of 5 × 10^3^ cells per well. After 24, 48, 72, and 96 h respectively, CCK-8 solution (10 µL, Dojindo, Kumamoto, Japan) was added into each well and the plates were incubated at 37 °C for 2 h. The absorbance value of each well was detected by a microplate reader (Thermo Fisher Scientific, Waltham, MA, USA) at 450 nm wave length.

### Flow cytometry

The transfected MC3T3-E1 cells were detached using trypsin (0.125%) and resuspended with phosphate-buffered saline (PBS). Cells were pelleted at 1000 rpm for 5 min and stained with Annexin V-FITC Apoptosis Detection Kits (BioVision, USA). Cell pellet was resuspended in 200 mL annexin V binding buffer, and the cells were counter-stained with 5 μL propidium iodide (PI) before analysis. Apoptotic cells were quantified by flow cytometry (BD Bioscience, USA), and the data were analyzed with Cell Quest software (Becton Dickinson, San Jose, USA).

### Western blotting

Tissues were lysed in RIPA buffer containing protease inhibitors. Proteins were resolved on 12.5% SDS-PAGE gels and transferred to PVDF membranes. Membranes were blocked in 5% non-fat milk at 37 °C for 2 h and probed with anti-Bcl-2 (ab32124, Abcam), anti-Bax (ab32503, Abcam), anti-cleaved-caspase-3 (ab32042, Abcam), and anti-Syne1 (ab192234, Abcam) primary antibodies (1:1000 dilution) overnight at 4 °C. β-actin was probed as a loading control. Membranes were washed and labeled with HRP-conjugated secondary antibodies. Blots were imaged using ECL advance western blotting reagent for chemiluminescence detection.

### qRT-PCR analysis

Total RNA was extracted from transfected cells or tissues using TRIzol reagent (Invitrogen, USA). cDNA synthesis was performed using Primescript RT Reagent (TaKaRa, Tokyo, Japan). RT-qPCRs were performed using SYBR®Premix Ex Taq™ Reagent (TaKaRa, Japan) and the StepOne Plus Real-Time PCR system (Applied Biosystems, USA). GAPDH was used as an internal control. Relative gene expression was calculated using the 2^−ΔΔCt^ method. Primers for RT-PCR were as follows: GAPDH (F: 5′-TGTGTCCGTCGTGGATCTGA-3′, R: 5′-TTGCTGTTGAAGTCGCAGGAG-3′), Runx2 (F: 5′-GAACCAAGAAGGCACAGACAGA-3′, R: 5′-GGCGGGACACCTACTCTCATAC -3′), Bglap (F: 5′-GACCGCCTACAAACGCATCTA-3′, R: 5′-CAGAGAGAGAGGACAGGGAGGA-3′), Col1a1 (F: 5′-GACATGTTCAGCTTTGTGGACCTC-3′, R: 5′-GGGACCCTTAGGCCATTGTGTA-3′), and ALP (F: 5′-GCAGTATGAATTGAATCGGAACAAC-3′, R: 5′-ATGGCCTGGTCCATCTCCAC-3′).

### Hoechst staining

MC3T3-E1 cells were stained with Hoechst 33258 staining solution (94403, Sigma-Aldrich) after fixation in 4% paraformaldehyde. Images were obtained on an Olympus fluorescence microscope (Olympus Corporation, Japan).

### Alizarin red staining

MC3T3-E1 cells were seeded into 12-well plates and cultured in osteogenic medium for 21 days. Cells were fixed in 70% ethanol on ice for 1 h, washed in ddH_2_O and stained with 40 mM Alizarin red S (Sigma-Aldrich, Missouri, USA) at a pH = 4.2 for 15 min. Cells were washed in ddH_2_O and PBS. Representative images were acquired using a confocal microscope (Olympus BX51) equipped with a digital camera (C2si confocal microscope, Nikon Corporation). To quantify the calcified matrix, 200 μL of 5% perchloric acid (Cat. 244252; Sigma-Aldrich) was added to each well and absorbance was measured at 420 nm using a microplate spectrophotometer.

### RNA-FISH

RNA-FISH was used to assay the localization of lncRNA NONMMUT004552.2 and miR-15b-5p in MC3T3-E1 cells. Briefly, cells were fixed in 4% paraformaldehyde for 20 min at room temperature, the cells were prehybridized with a hybridization solution. Then, lncRNA NONMMUT004552.2 and miR-15b-5p were labeled with CY3 and FAM fluorophores, respectively. Nuclei were stained with DAPI. Cells were imaged on a confocal microscope (FV1000, Olympus, Japan).

### Cyto-Immunofluorescence

MC3T3-E1 cells were washed in PBS and fixed in 4% paraformaldehyde for 15 min. Cells were permeabilized with Triton X-100 (0.025%) for 10 min, blocked in 1% goat serum for 1 h and probed with primary antibody anti-Syne1 (1:100; ab192234, Abcam) at 4 °C overnight. Cells were washed and labeled with FITC-conjugated secondary antibody (ab8211, Abcam) for 1 h. Nuclei were stained with DAPI for 10 min and imaged on a confocal microscope (FV1000, Olympus, Japan).

### ALP activity and labeling

Supernatants from MC3T3-E1 cells were centrifuged at 12,000 rpm for 15 min and ALP activity measured using commercial kit (ALP assay kit: Nanjing Jiancheng Technological Inc., China). Values were normalized to protein content through BCA protein assay kit (Thermo Fisher Scientific, USA). For ALP staining, cells were fixed with 4% paraformaldehyde (Sigma, Shanghai, China) for 15 min at room temperature and washed three times with phosphate-buffered saline (PBS). ALP was labeled in fixed cells using NBT/BCIP staining kits (Beyotime Biotechnology, China). Cells were imaged on a digital camera (Coolpix 4500, Nikon, Tokyo, Japan).

### Statistical analysis

Data are expressed as the mean ± SD. Statistical analysis was performed using GraphPad Prism 7.0. All data for cell analysis were generated from three independent replicates. For animal analyses, six mice were assigned to each experimental group. Prior to the statistical analysis, the comparison data were normally distributed. Single group comparisons were performed using a Student’s *t*-test. Multiple group comparisons were performed using two-way ANOVA or one-way ANOVA followed by Tukey’s posthoc test. *P* < 0.05 was deemed significant.

### Supplementary information

Table S1

## Data Availability

All the data used to support the findings of this study are included within the article and its supplementary information files.

## Code availability

No custom algorithm, software, or code was used in the present work.
