# Supplementary material for: Long non-coding RNA-NONMMMUT004552.2 regulates the unloading-induced bone loss through the miRNA-15b-5p/Syne1 in mice
Source: NPJ Microgravity. 2024 Mar 23;10:37. doi: 10.1038/s41526-024-00382-8 (PMC10960867; doi:10.1038/s41526-024-00382-8)
Supplement: Supplementary file 1 — Table S1 [file 41526_2024_382_MOESM1_ESM.docx]

**Table S1 The information of lncRNA-NONMMUT004552.2**

| NONCODE TRANSCRIPT ID | [NONMMUT004552.2](http://www.noncode.org/cgi-bin/hgTracks?position=chr10:5025380-5029003&nonLnc=full&hgFind.matches=NONMMUT004552.2&PhyloNONCODEGene=hide&PhyloNONCODELncRNA=hide&db=mm10) |
| --- | --- |
| NONCODE Gene ID | [NONMMUG002928.2](http://www.noncode.org/show_gene.php?id=NONMMUG002928&version=2&utd=1) |
| Chromosome | chr10 |
| Start Site | 5025380 |
| End Site | 5029003 |
| Strand | - |
| Exon Number | 1 |
| CNCI Score | -0.0983040 |
| Length | 3623 |

Sequence

>NONMMUT004552.2

ATTGTATTATCTAATTCCCCTTCTCCCACAAATGAATTGTGGATGTTCCGTGACCAGAGGTGAAGCCATCCCACAGCAGTCCTTTGAGAAAGCCACAGATCCAGTCCTCTGACTGACAAAAGGGATGTTGCTAATAAGGGAGGAAGAAGTTAGATGGCAGCAAGAGTAGCCATTGGATCACAAATACCAAATCTATCTGCTCCCCTAATTAGTTTAGGCTGCCACCTAGGCGTTCTGAATGGCTAACATGTTGTCTCTTAAGGCATCAGTTGCTTTCATGTGCCTAACAGGTCTTTTACCAATGTGCCTGCCACATTCCCGCTGCAATACTCACGAAGCTCCTATCGGCTCTATTCTGCCCAGAATCGCTAATTAACACTCCTCTGAAAGTTCACCAGTGCTCTCTCGGGTCCCTTGGGAGGGAATCGATTGCAAGGAGGCTTTACCTGGGTGACATGCTCCATCAAACACCTTGGCAAACGAGCTCCTCAACTCCAGGGAACCTTGGATCAGGGGCACTCAGCCCTGTCAATGGGCACAGAGGGGATGTTTGCCTCACTTACAGTCTAGGCAGCTATCCATTCTTTGGCACAGAAGCTGACCTTGTGTTTGATCTTGCACTGCTCCAACAAATCTGGAAGGTTACCGAAGAAGCACGGGATTTAGAGACAGATGCTATCTTCTGAAAACAAGAAGGAGCTAGTTTGGGAAGGTATTGGTTTAAAGGGAGAGGTTGTATTTTGTTGTGGTTTTCAACTAAATGGTAACAACATCCTCACCTGTTATATATTTCAGTTGAAATTTAAGTTGGCAATTCCATGTTCTCATTGATTATTACAATAGTCTAATTGGATGGAGGCCTGTTACTCAGAGTCTCTTTAGGCCCCAAATCTACAAAATCAATACATGATGCTTTCTTCCATTAAGAGATATGAGATCCAGAGATTCCTAGAATCAATTACCAAGAGCTTCCAAAGAACTTGCTAAGATTATAAGAAACCAAAAATACAAAATAAAACGGTGCCTGCATTTATACTGCCCCATAATACTACTGGGTTTCATTTGAGGTTAAGCACATAGAAGTGTGCCTTCAGAACAGTGCTGTTTGTGGTCCCAGCATCTGACATAAGATTCTGTAACCCCGTCACCAAAAGTCTTCCATACAGATATGCACGGATGAGCAACAGTTTAAGAAACTGGGAAGGTGATGTGCTAGTTTTCAGCAAGATAGTAGACTGTGATTCTCTTCACCCTGTGTGGGATGAAGGATTACTTCACTCTACAGCTGAGAACACAGAGAGGCAGAACTTTTCTGGATGCTTTTAGTTTTGGCCTCTTCTGCACAAAGAATTAAGAAGGAATAGGAGCCAAAAAGGACTAGCTGAAGTCCTGGTAGGAGGCCCAAGAAAGAGGGGTCCTTTTAAATTGAAAATTTCAAATTTCAAAGGTAGTCAAATACATTTAAGCAAGCCAGTAGGGGGTTTCTTTTGCTTGTTCACTAAGGGACTCCTAAAGGTGCTGTTCAATTGATTGTGAAATTTGTTTTCCAATTTCTTCAATTGGACTCTTCCTGGAAGCCCATTGTTCAAAGCATCCAAAGAGCCTTAGCATAAAGCAGTTGCCCTTGGAGAGGCAGCTGCATTCATGCCTGAAGACAACTGTAGGGTGGGGCACCAACAGGCTTCTCTTTTGCTCTTCTGAAGTGGGATGTCCCAGTGTTTGGGAAATAATATCTTTGAAACCACTGTTAGTAAGTCAGAAATGTTTTCCCGAGAGAACAGACTGAATTCAGCAGTTTTCAAGGATATTACTCAGCACACCCCAGTATAGCCATACAGAGAACAACTGTTAGGTgctggcatttatctagtacttaatgtgttttgagcaccatgctaagaattgtgtgtgttacctcagttaacatccctgtgatatggctggctagatccctcaacttacagacaagaaCAAGAGATTAGAAATATGAACACAGGTCTCACAAGACTCTGAACCCTACTATACTGAAAAGACACCTCTCTCCCACTGGGATGTCCATAGTAGGATTAATCAAGCAATGTATGCATAGGACCAGGTGAACTTTCCATTTGGAAGGAGGGAATAACAGAGCCCTCAGTTGCTTCTTGCAACTTGCCTTCTCAGCACACAGCTAGGAGCTACTGAATGTCAAACACTATAGATGGACAGCTAGAAATTTCTGCATATGGAATTTATGTATCCTGTCTAACAAGTGTCAAAACAAACTGAAAATATCCCCCCCAGCTTTGTAAGATGTACACATGTAATTATCTCATTATATCTTCCTTGACCCTGTGGGATCTGCAAACTGAGTTACCTACTATATGCAGATCCCCAGGCCTCAGGGTTCTCTTTGTTATATGCACTTTCTTTTGTGTCATTTGGTTTTAGACCCTGGTCCATCCCTTACTTTCCTCCTTCTTCCATGAGTATGTGTTCTAGAAAAGGGACAAACAGTTATTTTTCTGCCAATCTGAAAGAAATAGAAGAGAAAGATGAGATAGAGATTTACTTAGAGAGAATATGCACATGTTATTCTGGACAAAATTACTTAAAATATCCCTATTGATTGAGTTCTTTGGGTACAGCCACACAGTTAAGTGTTCCCATACAAATGCAGTCTACACTGTCACCCTTGTCTATACAGAAGCCATTGAAGAATGGCCATGAGCAAGGCCACTTATCACTATTGCTCTGTCTTCTATTGAATTTTTCAGTACTCTTGAAAATTGGATGACCTTAAGTCAGTAAGTTAATATTAAAATAAGCACCCTTTTACTCTGAGATGATTAACCTATTGGCGCTAAAATCTCTAAGGCAGAGTCACCCTGATGGAGCAACTTTGAAACTCTGAGAATTCTTACAGTTACTTCAGGTTGAGTGAGCACTTGTCTCAGTAGGTAGACTGGACATTTAGTCCAATTGCACTGATAACCACTAAGTACTGTGTGTTCTCTTTGTCCCTGCCTCCTCAGCCCATTACTTCTCGCTAAGTTGGCATCAACAGTAGAAGTCAGGTGGCGGTTACATTTTACTAAAGAACGTTCTGATGTGGTTAAATGTGCCATGTCCTCCAAAGGATTCTGTCACATGTGAGCCCATGCTCTCCAAATGATAATCTCTCTAGTTCTTGGTAGGAATGAACTGGTCATGACAAAACCTCTGGGTTTTTTTTGTTTTTTTGTTTTTTTTATATCCTGTCCTTTAACCTCACAGAAGCTAATAATTGACAAAGAGGAGCTATCCAGCTCCATTTGATTGAATTCTTAATTGTTTTGTGGACCTTTTCCCATCCCTAAAGTATCTAAATATCTCCTGGTTGAAGTGGTCTTGAGGAATGGAGCTTCATCTTCAGCCATTTATCCAGGGTCAGTGCTGTTTGCCAGGGACTATTTTCACATCAACATGTACTCAAGGATCATGCCAAAGAAAAACCCAGTACAGAGTCTGCCCTCCCCTTCTATCTCATATTTCTCAGGACACTTTAAAACAAACAAACTATTGAGTAAGCCATCAAAGAAAAAATGACTTTGGTACTTAATATGCTATGCCATCCGTCTTCTTTGAAAACAATGAAAGGAACC
